# circEVI5 acts as a miR-4793-3p sponge to suppress the proliferation of gastric cancer

**DOI:** 10.1038/s41419-021-04061-4

**Published:** 2021-08-05

**Authors:** Meinan Yan, Liling Niu, Jing Liu, Yuan Yao, Hui Li

**Affiliations:** 1 Department of Gastrointestinal Cancer Biology, Tianjin Medical University Cancer Institute and Hospital, Tianjin, China; 2 Key Laboratory of Cancer Immunology and Biotherapy, Tianjin, China; 3 National Clinical Research Center for Cancer, Tianjin, China

**Keywords:** Gastric cancer, Oncogenesis

## Abstract

Circular RNAs (circRNAs) are a novel class of endogenous noncoding RNAs (ncRNAs) with a covalently closed loop structure. Accumulating evidence shows that circRNAs play vital roles in the growth, metastasis, treatment and prognosis of various cancers. However, the detailed functions and underlying mechanisms of circEVI5 (hsa_circ_0013162) in gastric cancer (GC) remain undocumented. In this study, the expression levels and prognostic value of circEVI5 were validated in GC tissue samples by using qRT-PCR. circEVI5 was significantly downregulated in GC tissues and cells, and low circEVI5 expression was correlated with poor prognosis. Next, in vitro CCK-8 assay, EdU incorporation assay, PI staining cell cycle assay, and in vivo xenograft mouse models were conducted to assess the functions of circEVI5. Gain of function experiments indicated that circEVI5 could inhibit GC cell proliferation and retard the cell cycle. Moreover, bioinformatics prediction showed that circEVI5 binds to miR-4793-3p, while FOXO1 may be a target of miR-4793-3p. Pull-down assays, RNA immunoprecipitation (RIP) assays, luciferase assays, and western blot were used to confirm the interactions between circEVI5, miR-4793-3p, and FOXO1. Functional assays demonstrated that circEVI5 suppressed the proliferation of GC by sponging miR-4793-3p and increasing FOXO1 expression levels. In conclusion, our study demonstrated that circEVI5 can bind miR-4793-3p as a ceRNA to eliminate the negative regulation of FOXO1, therefore suppressing GC proliferation.

## Introduction

Gastric cancer is still a major threat to human health [1]. Based on global cancer statistics [2], GC is the fifth most diagnosed malignancy and the fourth most common cause of cancer-related death in the world, and thus, it is necessary to explore GC-specific biomarkers and underlying molecular mechanisms to improve diagnosis and therapies.

CircRNAs are a new subgroup of ncRNAs. Previously, circRNAs were found and considered byproducts of aberrant splicing events [3]. However, with the rapid development and application of high-throughput RNA sequencing technology [4], the field of circRNAs has become increasingly clearer. CircRNAs are widely present in eukaryotic cells [5], and most of them are highly conserved across species [6]. CircRNAs are formed by back‐splicing events [7], which lead to covalently closed RNA loops with a unique head‐to‐tail junction. Due to the lack of the typical 5′ cap and poly (A) tail, circRNAs escape ribonuclease digestion [8] and eventually accumulate in certain tissues [9] or body fluids [10]. These features indicate important functions of circRNAs in human diseases.

Currently, the roles of circRNAs have been experimentally confirmed in multiple pathological processes, such as diabetes [11], Alzheimer’s disease [12], atherosclerosis [13], and especially in the progression of human cancer [14]. Studies have found diverse biological functions of circRNAs, and acting as miRNA sponges may be the main mechanism [15]. CircRNAs have multiple miRNA response elements (MERs), which can act as competitive endogenous RNAs (ceRNAs) to bind miRNAs and modulate downstream gene levels [15]. For example, ciRS-7 was first demonstrated to harbor 63 conserved binding sites for miR-7 [16], and overexpression of ciRS-7 could exert the suppressive effects of miR-7 on several oncogenes [17, 18]. In addition, circRNAs can also regulate the activity of proteins [19] or the expression of host proteins [20] by serving as protein sponges or decoys. CircFOXO3, which is highly expressed in noncancer cells, can form ternary complexes with p21 and CDK2 to retard cell cycle progression [21]. In addition, circRNAs could be translated into proteins in some cases and there is strong evidence that circRNAs which contain the open reading frame (ORF) can encode proteins by initiating the internal ribosome entry site (IRES) [22]. To date, aberrant expression of circRNAs has been found in many cancer tissues [23, 24], thus, circRNAs have the potential to be promising molecular biomarkers and new therapeutic targets.

We previously explored the circRNA expression signatures using an RNA-seq analysis from GC patients with different prognosis. A novel circular RNA termed circEVI5 (according to circBase (http://www.circbase.org/), also annotated as hsa_circ_0013162) which significantly upregulated in GC patients with good prognosis, attracted our attention. In this study, we explored the role of circEVI5 in the development and progression of GC. circEVI5 was significantly downregulated in GC tissues as well as GC cell lines, and low circEVI5 expression was correlated with poor prognosis. circEVI5 could act as a sponge of miR-4793-3p to regulate the expression of FOXO1, and further retard the cell cycle and inhibit the proliferation of GC cells. Our findings reveal a promising target for the diagnosis and treatment of GC.

## Results

### circEVI5 is downregulated in gastric cancer, and low circEVI5 expression correlates with poor prognosis

**Fig. 1:**
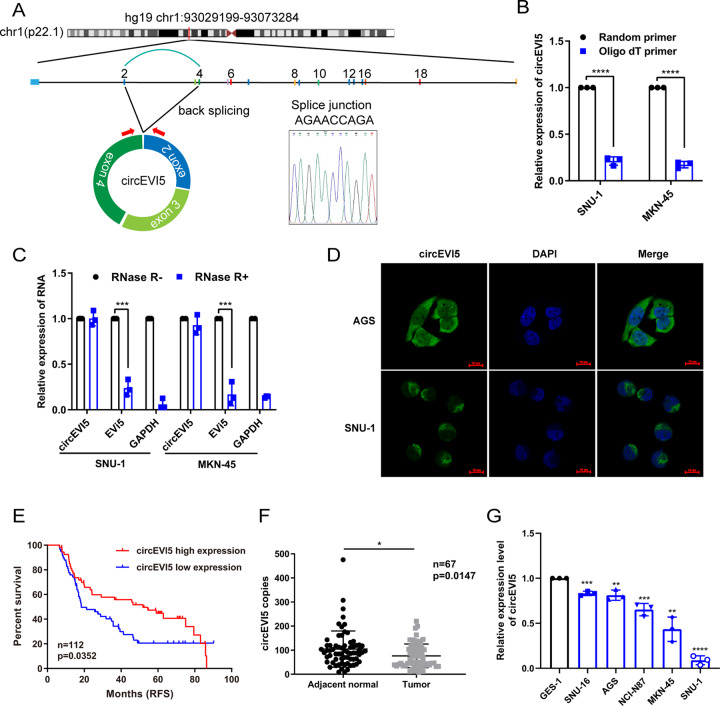
circEV15 was downregulated in GC, and low circEV15 expression was correlated with poor prognosis. **A** Schematic illustration of the formation of circEVI5. Sanger sequencing confirmed the back splice site of circEVI5. **B** The template cDNA of circEVI5 reverse transcribed by random primers or oligo dT primers was analyzed by qRT-PCR in SNU-1 and MKN-45 cells. **C** qRT-PCR analysis of circEVI5 expression in SNU-1 and MKN-45 cells treated with or without RNase R. **D** FISH assay showed circEVI5 (green) localized in the cytoplasm, and nuclei were stained with DAPI (blue), scale bar, 10 μm. **E** The RFS curve was drawn using the Kaplan-Meier method (*n* = 112). Low circEVI5 expression showed a poor RFS (*p* = 0.0352). **F** circEVI5 expression was measured by qRT-PCR in 67 pairs of GC (Tumor) and matched normal tissues (Adjacent normal). **G** The expression levels of circEVI5 in GC cell lines were evaluated by qRT-PCR. Data were shown as the mean ± SD of three independent experiments. *p < 0.05, **p < 0.01, ***p < 0.001, ****p < 0.0001.

circEVI5 is derived from exon 2, 3, and 4 regions within the EVi5 (ecotropic viral integration site 5) locus (human hg19 chr1:93029198-93073284), which is located on chromosome 1p22, and the spliced length is 339 bp. The head-to-tail splicing of exon 2 and exon 4 was confirmed by Sanger sequencing (Fig. 1A). Since circRNAs lack a poly (A) tail, we used random primers or oligo dT primers for RT-PCR. circEVI5 reverse transcribed by oligo dT primers was much less than by random primers (Fig. 1B). Moreover, circEVI5 also displayed resistance to RNase R digestion, while EVI5 linear RNA was degraded (Fig. 1C). In addition, FISH assays illustrated the predominant cytoplasmic localization of circEVI5 (Fig. 1D).

To analyze the correlation between the levels of circEVI5 and clinicopathological features and prognosis, we collected the clinical data of the 112 GC patients. As shown in Table 1, the expression level of circEVI5 negatively correlated with tumor size of GC (*p* < 0.05). Additionally, in Kaplan–Meier analysis, patients who had lower expression of circEVI5 within their GC tissues had significantly reduced relapse-free survival (RFS) rate (Fig. 1E). We further validated the expression of circEVI5 in 67 paired GC and adjacent normal tissue samples and found that the expression of circEVI5 was significantly lower in GC tissues compared with adjacent normal tissues (Fig. 1F). Meanwhile, the expression level of circEVI5 in various GC cells was also lower than that in regular gastric epithelial GES-1 cells (Fig. 1G). All of these results suggested that circEVI5 was a stable and cytoplasmic circRNA that might be an appropriate biomarker.

**Table 1:** Relationship between circEVI5 expression and clinicopathologic factors of patients with gastric cancer.

Parameter	No. of patients	circEVI5 (high)	circEVI5 (low)	*p*-value
***Sex***				
male	90	38	52	0.7836
female	22	10	12	
***Age (year)***				
> 60	60	23	37	0.2987
≤ 60	52	25	27	
***Tumor size (cm)***				
> 6	52	17	35	0.043*
≤ 6	60	31	29	
***Differentiation grade***				
Moderate-differentiation	51	18	33	0.1392
Poor-differentiation	61	30	31	
***T stage***				
T1–T3	14	8	6	0.2482
T4	98	40	58	
***N stage***				
N0–N1	58	29	29	0.1134
N2–N3	54	19	35	
***Lymph node status***				
Negative	28	15	13	0.1859
Positive	84	33	51	
***TNM stage***				
I–II	29	15	14	0.2623
III–IV	83	33	50	

The TNM Staging System is based on the tumor (T), the extent of spread to the lymph nodes (N), and the presence of metastasis (M).

*p < 0.05.

### circEVI5 suppresses cell proliferation and blocks G1 to S cell cycle transition in gastric cancer cells

**Fig. 2:**
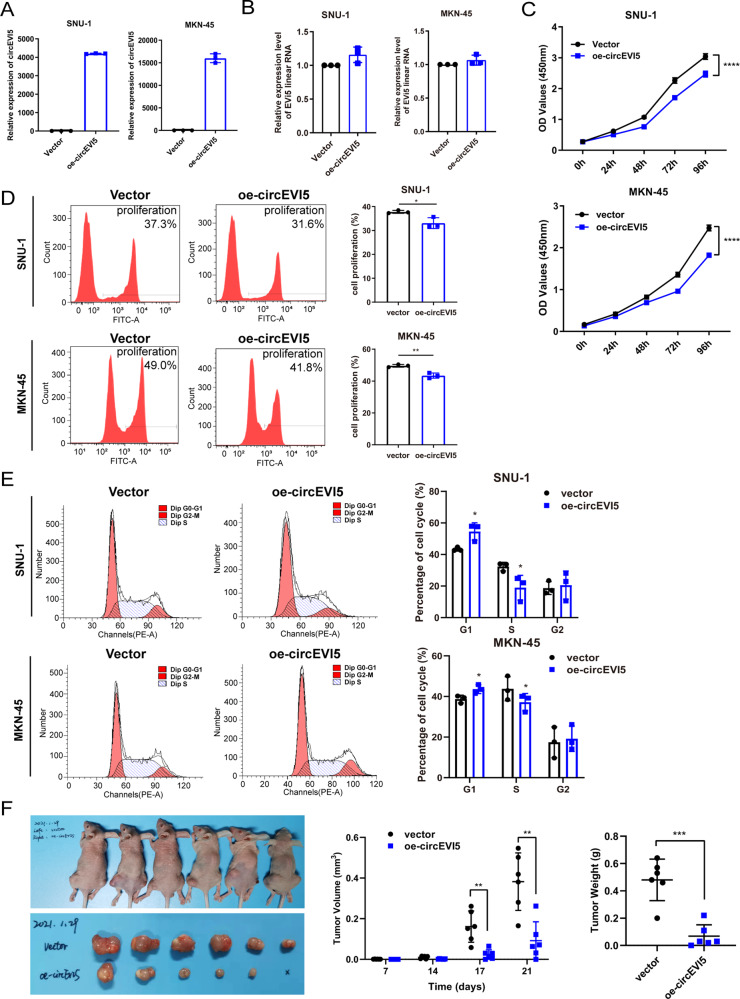
circEVI5 suppressed cell proliferation and blocked cell cycle progression in GC cells. **A**–**B** The expression of circEVI5 and EVi5 linear RNA was verified by qRT-PCR after stable transfection with circEVI5 or control vector in SNU-1 and MKN-45 cells. **C**–**D** The CCK-8 assay and EdU incorporation assay were performed to detect the proliferation of SNU-1 and MKN-45 cells transfected with circEVI5 or control vector. **E** Representative images of PI-stained cell cycle analysis by using flow cytometry. **F** Xenograft models were built by inoculating circEVI5 or control vector cells subcutaneously into the backs of BALB/c nude mice, *n* = 6. The tumor volume was periodically measured and tumor growth data points were plotted. After 21 days, the mice were euthanized, and tumor tissues were excised and weighed. Overexpression of circEVI5 is represented as oe-circEVI5. Data were shown as the mean ± SD of three independent experiments. *p < 0.05, **p < 0.01, ***p < 0.001, ****p < 0.0001.

Considering the low expression of circEVI5 in GC, we investigated the function and downstream regulatory pathway of circEVI5 through the stable overexpression of circEVI5 in GC cells. Obvious overexpression of circEVI5 was observed after transfection (Fig. 2A), while no significant effect was found on its parental gene EVi5 (Fig. 2B). As indicated by the functional experimental results, the viability of SNU-1 and MKN-45 cells with overexpressed circEVI5 was significantly inhibited (Fig. 2C). Meanwhile, DNA synthesis of SNU-1 and MKN-45 cells was retarded by circEVI5 overexpression (Fig. 2D). In addition, flow cytometry analysis showed that more cells with overexpressed circEVI5 were distributed in the G1 phase, revealing that circEVI5 overexpression could block cell cycle progression at the G1 to S phase (Fig. 2E). We further elucidated the function of circEVI5 in xenograft mouse models bearing tumors originating from MKN-45 cells transfected with circEVI5 or control vector (*n* = 6). After 21 days of monitoring, we found that overexpressing of circEVI5 generated a dramatically negative effect on the volume and weight of the xenograft tumors (Fig. 2F). Collectively, circEVI5 could inhibit the proliferation of GC cells in vitro and in vivo.

### circEVI5 serves as a miR-4793-3p sponge

**Fig. 3:**
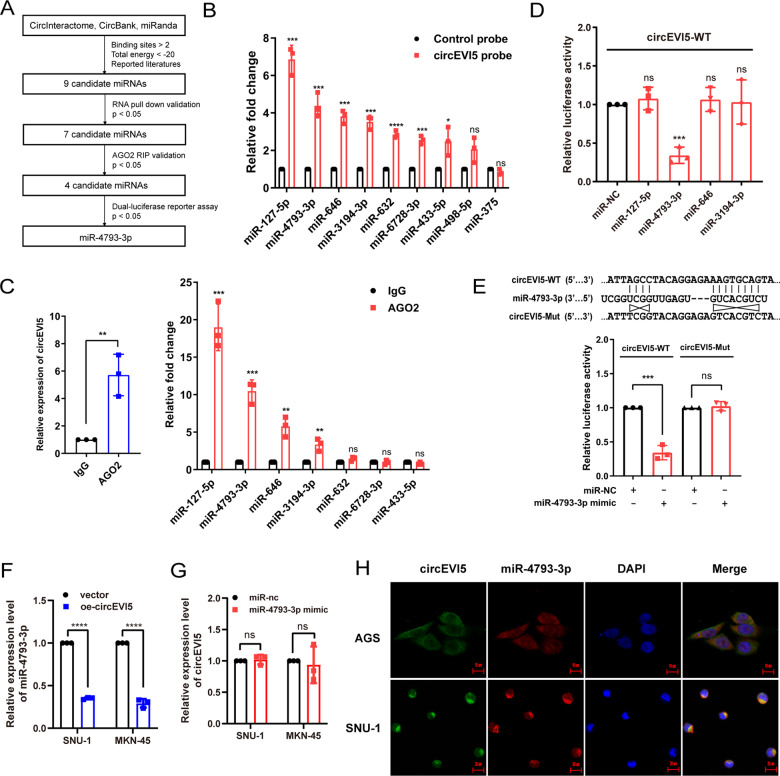
circEVI5 served as a miR-4793-3p sponge. **A** The flow chart illustrated the screening criteria of identifying miR-4793-3p as the target of circEVI5. (The lower energy value indicates that higher energy is required for the disassembly of the miRNA-circRNA interaction, indicating a more reliable conjugation relationship.) **B** QRT-PCR confirmed the enrichment of miRNAs pulled down from the circEVI5 probe. miR-127-5p, miR-4793-3p, miR-646, miR-3194-3p, miR-632, miR-6728-3p, and miR-433-5p were statistically significant enriched (*p* < 0.05). **C** The RIP assay was executed to detect circEVI5 and miRNAs captured by anti-AGO2. miR-127-5p, miR-4793-3p, miR-646, and miR-3194-3p were statistically significant enriched (*p* < 0.01). **D** The relative luciferase activities were detected in HEK-293T cells after co-transfection with circEVI5-WT dual-luciferase reporter and miR-127-5p, miR-4793-3p, miR-646 or miR-3194-3p mimics, respectively. **E** The relative luciferase activities were detected in HEK-293T cells after co-transfection with circEVI5-WT or circEVI5-Mut dual-luciferase reporter and miR-4793-3p mimics or miR-NC, respectively. **F** The expressions of miR-4793-3p were analyzed by using qRT-qPCR in cells transfected with circEVI5 or control vector. **G** The expression levels of circEVI5 were determined with qRT-qPCR in cells transfected with miR-4793-3p mimics or miR-NC. **H** The FISH assay was performed to observe the colocalization of circEVI5 (green) and miR-4793-3p (red) in the cytoplasm, and nuclei were stained with DAPI (blue). Scale bars, AGS: 10 μm, SNU-1: 20 μm. Data were shown as the mean ± SD of three independent experiments. *p < 0.05, **p < 0.01, ***p < 0.001, ****p < 0.0001.

It has been extensively reported that circRNAs can act as miRNA sponges and regulate downstream genes [25], thus, we predicted potential miRNAs that bind to circEVI5 by using CircInteractome, CircBank, and miRanda, as well as the results reported in the literature. Nine candidate miRNAs were obtained and evaluated in subsequent validation assays (Fig. 3A). Firstly, in the RNA pull-down assay, seven miRNAs (miR-127-5p, miR-4793-3p, miR-646, miR-3194-3p, miR-632, miR-6728-3p, and miR-433-5p) were statistically significant enriched by the specific circEVI5 probe (*p* < 0.05) compared with the negative control probe (Fig. 3B). Secondly, given that AGO2 is the core protein of the RNA-induced silencing complex (RISC) [26], we measured the protein-RNA complexes from the anti-AGO2 RIP assay by qRT-PCR. The results demonstrated that endogenous circEVI5 and four markedly enriched miRNAs (miR-127-5p, miR-4793-3p, miR-646, and miR-3194-3p) were successfully captured by the AGO2 antibody (*p* < 0.01) compared with IgG antibody as the negative control (Fig. 3C). Thirdly, to find evidence of direct binding between circEVI5 and target miRNAs based on their complementary sequences, four aforementioned miRNAs were co-transfected with the wild-type circEVI5 dual-luciferase reporter, respectively. An obvious reduction of approximately 60% in luciferase reporter activity was observed only when wild-type circEVI5 and miR-4793-3p mimics were co-transfected (Fig. 3D). However, no significant change was observed in luciferase reporter activity after co-transfection of miR-4793-3p mimics and the mutant circEVI5 dual-luciferase reporter (Fig. 3E). QRT-PCR further confirmed that circEVI5 overexpression could decrease the miR-4793-3p levels (Fig. 3F), while miR-4793-3p failed to influence circEVI5 levels (Fig. 3G). Furthermore, the FISH assay illustrated that circEVI5 and miR-4793-3p were colocalized in the cytoplasm (Fig. 3H). Taken together, these combined evidences strongly suggested that circEVI5 could act as a miR-4793-3p sponge.

### miR-4793-3p can reverse the repression function of circEVI5 in GC cells

**Fig. 4:**
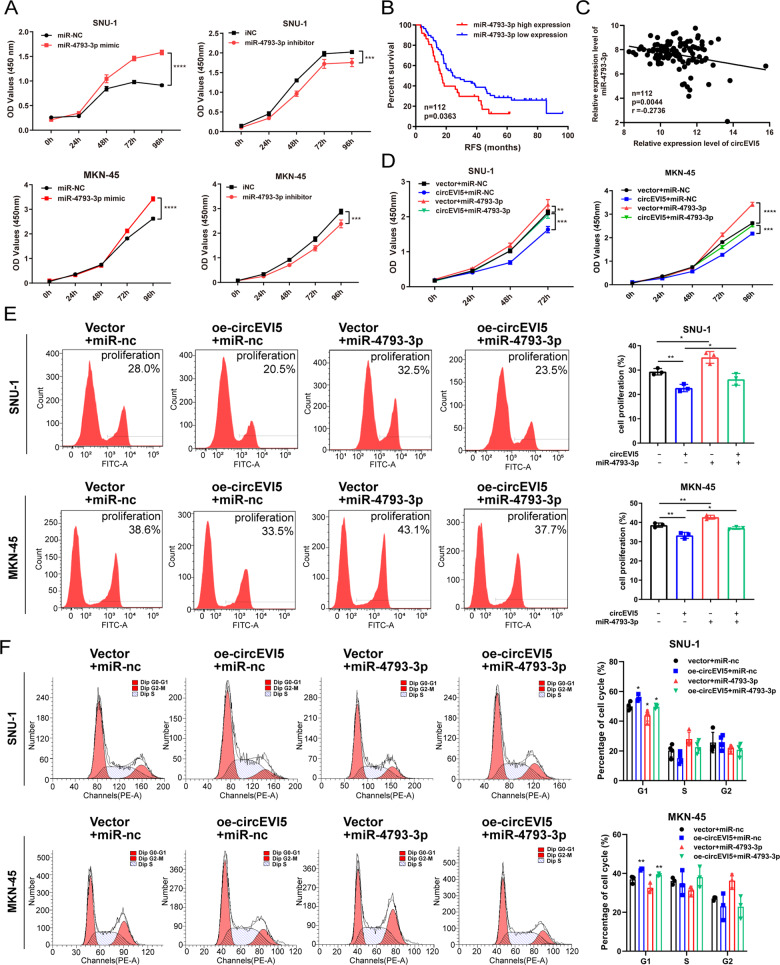
miR-4793-3p could promote GC cell proliferation and reverse the effects of circEVI5 on GC cells. **A** The proliferation ability of SNU-1 and MKN-45 cells transfected with miR-4793-3p mimics or inhibitors were detected by the CCK-8 assay. **B** The RFS curve was drawn using the Kaplan–Meier method (*n* = 112). High miR-4793-3p expression showed a poor RFS (*p* = 0.0363). **C** Pearson correlation analysis determined the significant negative correlation between the levels of circEVI5 and miR-4793-3p in 112 GC tissues (*p* < 0.01). **D**–**F**. The CCK-8 assay (**D**), EdU incorporation assay (**E**), and PI staining cell cycle assay (**F**) were applied for rescue experiments in SNU-1 and MKN-45 cells to determine whether the biological function of circEVI5 in GC cells could be affected by miR-4793-3p. Rescue experiments were performed with upregulated miR-4793-3p on the basis of circEVI5 overexpression. Overexpression of circEVI5 is represented as oe-circEVI5. Data were shown as the mean ± SD of three independent experiments. *p < 0.05, **p < 0.01, ***p < 0.001, ****p < 0.0001.

Based on the interaction between circEVI5 and miR-4793-3p, we next evaluated the potential functional roles of miR-4793-3p by transfecting miRNA mimics or inhibitors into SNU-1 and MKN-45 cells. The CCK-8 assay indicated that miR-4793-3p could promote the proliferation of GC cells (Fig. 4A) and the Kaplan-Meier analysis showed that GC patients with high miR-4793-3p expression had a shorter RFS (Fig. 4B), indicating miR-4793-3p may act as a tumor suppressor and high miR-4793-3p predicts a poor prognosis in GC. Pearson correlation analysis showed that the expression level of circEVI5 negatively correlated with miR-4793-3p in GC tissues (*p* < 0.01) (Fig. 4C). To further determine whether circEVI5 exerts its biological function by sponging miR-4793-3p, the CCK-8 assay, EdU incorporation assay, and PI staining cell cycle assay were applied for rescue experiments. We observed that when circEVI5 and miR-4793-3p mimics were co-transfected, the reduced cell viability and EdU incorporation rate mediated by circEVI5 overexpression could be reversed (Fig. 4D, E), and the blocking effect of circEVI5 on GC cell cycle progression at the G1/S phase was counteracted (Fig. 4F). These results revealed that circEVI5 promotes GC cell proliferation by sponging miR-4793-3p.

### circEVI5 competitively binds to miR-4793-3p as a ceRNA to upregulate FOXO1 expression

**Fig. 5:**
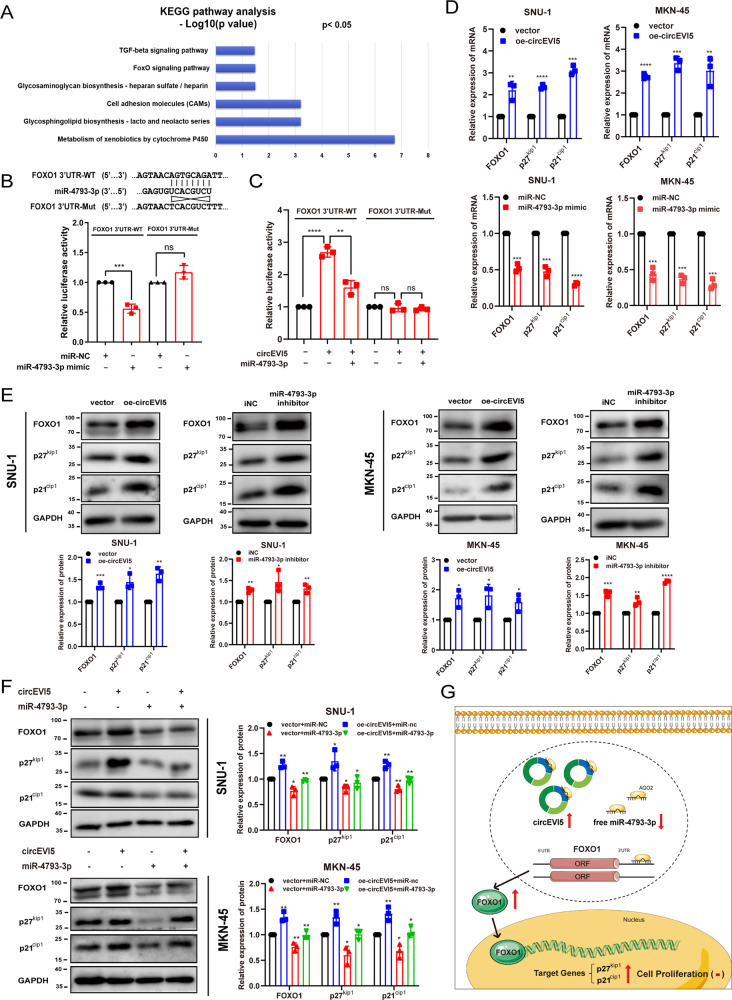
circEVI5 competitively bound miR-4793-3p as a ceRNA to upregulate FOXO1 expression. **A** Target genes of miR-4793-3p were predicted, and pathways were analyzed by using mirPath v.3 and microT_CDS. **B** The relative luciferase activities were detected in HEK-293T cells after co-transfection with FOXO1 3′UTR-WT or FOXO1 3′UTR-Mut dual-luciferase reporter and miR-4793-3p mimics or miR-NC, respectively. **C** The relative luciferase activities were analyzed in HEK-293T cells co-transfected with circEVI5 or control vector and miR-4793-3p mimics or miR-NC and FOXO1 3′UTR-WT or FOXO1 3′UTR-Mut dual-luciferase reporter. **D** Relative mRNA levels of FOXO1, p27^kip1^, and p21^cip1^ were evaluated by qRT-PCR in SNU-1 and MKN-45 cells transfected with circEVI5 or control vector or the miR-4793-3p mimics or miR-NC. **E** Pearson correlation analysis determined the significant negative correlation between the levels of miR-4793-3p and FOXO1 mRNA in 112 GC tissues (*p* < 0.01). **F**–**G**. Western blot of FOXO1, p27^kip1^, and p21^cip1^ levels in SNU-1 and MKN-45 cells with circEVI5 overexpression or miR-4793-3p inhibition. Rescue experiments were performed with upregulated miR-4793-3p on the basis of circEVI5 overexpression. GAPDH was used as a loading control. **H** A schematic for the circEVI5/miR-4793-3p/FOXO1 pathway. Overexpression of circEVI5 is represented as oe-circEVI5. Data were shown as the mean ± SD of three independent experiments. *p < 0.05, **p < 0.01, ***p < 0.001, ****p < 0.0001.

Target genes of miR-4793-3p were predicted, and pathways were analyzed by utilizing mirPath v.3 and microT_CDS. KEGG analysis indicated that miR-4793-3p might likely participate in the FOXO (forkhead box O) signaling pathway, consistant with our functional experiments results (Fig. 5A). In addition, previous studies have validated that FOXO1, a candidate gene, could regulate the transcriptional level of genes related to the cell cycle, such as p27^kip1^ and p21^cip1^ [27].

To confirm this hypothesis, a mutation was generated in the putative binding sites in the 3′UTR of FOXO1. Dual-luciferase reporter assays showed a significant reduction of approximately 50% in luciferase reporter activity only when wild-type FOXO1 3′UTR and miR-4793-3p mimics were co-transfected (Fig. 5B). In order to further explore the interaction among circEVI5, miR-4793-3p, and FOXO1, we analyzed FOXO1 3′UTR luciferase reporter activity upon circEVI5 modulation. The results showed that the overexpression of circEVI5 could significantly increase the activity of wild-type FOXO1 3′UTR luciferase reporter, and the co-transfection of circEVI5 and miR-4793-3p could eliminate this effect. However, these effects disappeared with the mutant FOXO1 3′UTR luciferase reporter (Fig. 5C). Moreover, we found that circEVI5 significantly increased the mRNA levels of FOXO1, p27^kip1^, and p21^cip1^, whereas miR-4793-3p could decrease the mRNA levels of FOXO1, p27^kip1^, and p21^cip1^ in SNU-1and MKN-45 cells (Fig. 5D). Pearson correlation analysis showed that the level of miR-4793-3p negatively correlated with the level of FOXO1 mRNA in GC tissues (*p* < 0.01) (Fig. 5E). Western blot analysis revealed the increased expression of FOXO1, p27^kip1^, and p21^cip1^ with circEVI5 or miR-4793-3p inhibitor transfection (Fig. 5F), while the expression of miR-4793-3p mimics counteracted this effect (Fig. 5G). These results suggested that FOXO1 might be a putative target of miR-4793-3p, and that circEVI5 could bind miR-4793-3p as a ceRNA to regulate the expression of FOXO1, p27^kip1^, and p21^cip1^ (Fig. 5H).

## Discussion

Given the high abundance, high stability, and tissue-specific features of circRNAs and their widespread presence in various body fluids, circRNAs show great potential as cancer biomarkers. We have previously analyzed the expression of circRNAs in GC tissues by RNA-seq. Here, circEVI5 attracted our attention. According to the clinical tissue samples test and subsequent survival analysis, a lower level of circEVI5 in GC patients had a significantly poor RFS, showing great prognostic value. Additionally, the greater clinical value of circRNAs has been explored. For example, hsa_circ_0000190, downregulated in GC, was significantly associated with the tumor diameter, lymphatic metastasis, TNM stage, and especially CA19-9 expression [28]. This may provide a new pattern that combines circRNAs with traditional biomarkers for diagnosis and prognosis, making circRNAs promising biomarkers.

We further verified the decreased expression of circEVI5 in GC tissues by using 67 matched primary GC tissues and paracarcinoma tissues. circEVI5 arises from the EVi5 gene and consists of the head-to-tail splicing of exon 2 and exon 4. EVi5 belongs to a small subfamily of Tre 2/Bub2/Cdc16 (TBC) domain-containing proteins [29]. Studies have shown that EVi5 can regulate the cell cycle by binding to Emi1 as a stabilizing factor, which maintains the abundance of Emi1 in the S/G2 phase and leads to the timely accumulation of cyclins [30]. This function is important for ensuring mitosis and genomic fidelity. Therefore, EVi5 can be a potential oncogene, and misregulation of Evi5 levels has been found in several cancer types [31, 32]. The interaction of Evi5 and Emi1 may indicate the involvement of Evi5 in tumorigenesis [33]. However, circRNA biogenesis is known to compete with pre-mRNA splicing, possibly resulting in a decreased level of linear mRNA with exon inclusion [7], and thus, we hypothesized that circEVI5 also has a regulatory role in the cell cycle, referring to its parent gene EVi5. Our experiments verified that circEVI5 could block cell cycle progression at the G1 to S phase, ultimately contributing to the inhibition of GC cell growth, highlighting that circEVI5 can be a candidate cancer suppressor molecule in GC.

In our study, we found that circEVi5 functions as a tumor suppressor via miR-4793-3p. miR-4793-3p could significantly facilitate GC cell vitality. Although only a few relevant studies mentioned miR-4793-3p, it was suspected to be a promoting factor for cancer progression. For instance, microarray analyses identified that miR-4793-3p was upregulated in the hepatic metastasis of colorectal cancer tissue samples [34] and in necrotizing enterocolitis, which may be involved in the TLR4 pathway [35]. SHU00238, an isoxazole derivative [36], promoted the apoptosis of colorectal cancer cells by downregulating miR-4793-3p levels [37]. Additionally, miR-4793-3p has been reported to be upregulated in patients with cirrhosis, and plays a role in the innate immune response by binding with Grem1 to suppress the TGF-β signaling pathway [38]. We are interested in exploring the detailed mechanisms of miR-4793-3p in fostering gastric cancer.

Growing evidence for circRNAs acting as ceRNAs makes it a universal functioning mechanism. Mature miRNAs are assembled into RISCs and recognize target genes by base-complementary pairing; then, the RISCs repress translation or degrade target genes [39]. Here, FOXO1 was verified to be a target gene of miR-4793-3p. FOXO1 was reported to be a tumor suppressor in a wide variety of cancers [40] via anti-proliferation [41], pro-apoptosis [42], or cell cycle arrest [43]. circEVI5 can competitively bind miR-4793-3p and upregulate FOXO1 levels, leading to an increase in p27^kip1^ and p21^cip1^ [27]. Since p27^kip1^ and p21^cip1^ are important negative regulators of the cell cycle, GC cell cycle arrest occurred in the G1 phase. CircRNAs release downstream molecules by competing with miRNAs, which not only participate in biological processes of tumor development but also have the potential to regulate tumor immunity. For example, miR-138 promoted the viability and invasion of CRC cells by targeting programmed death-ligand 1 (PD-L1), and PD-L1 was upregulated and interacted with PD-1 to help tumors evade immune surveillance. Nevertheless, hsa_circ_0020397 could antagonize the miR-138 suppression of cell growth.

Therefore, circRNAs serve as ceRNAs to mediate miRNA functions and can be developed as effective therapeutic targets. The most direct method is exogenous upregulation (plasmids and lentiviral vectors [44, 45]) or downregulation (siRNA [46] or CRISPR/Cas9 system [47]) of circRNAs to target relevant miRNAs. Recently, artificial circRNA sponges containing miR-21 binding sites were synthesized and assessed by using cell proliferation assays in GC cell lines [48]. Exosomes, lipid bilayer membrane-enclosed vesicles, might be effective deliverers by containing engineered circRNAs or siRNAs targeting circRNAs [49, 50]. This result proved that synthetic circRNA sponges could achieve the therapeutic loss of function targeted against specific miRNAs in vitro. However, the use of circRNAs as therapeutic targets has not been verified in clinical trials, and their safety and side effects are still unclear. More detailed and complete experimental data are needed for real clinical applications.

In conclusion, we identified a prognosis-related circular RNA termed circEVI5 that was downregulated in GC tissues and cell lines. circEVI5 markedly inhibited GC cell proliferation by competitively binding miR-4793-3p as a ceRNA to upregulate FOXO1. Our findings provide a novel potential biomarker for prognosis and a promising treatment target in gastric cancer.

## Materials and methods

### Tissue samples and clinical data

We retrospectively collected RNA specimens of 112 GC patient tissues from January 2010 to December 2012 as well as 67 paired cancer tissues and adjacent normal tissues from January 2014 to December 2015. All patients received radical surgical resection without adjuvant chemoradiotherapy before surgery, and the pathological diagnosis was gastric adenocarcinoma. The follow-up interval began on the date of surgery and ended on the date of disease progression, death, or the last clinical investigation. Clinicopathological features, which included sex, age, tumour size, differentiation grade, T stage, N stage, lymph node status, and TNM stage (American Joint Committee on Cancer classification, AJCC) are shown in Table 1. The Medical Ethical Committee of Tianjin Medical University Cancer Institute and Hospital approved this study.

### Quantitative real-time PCR (qRT-PCR)

Total RNA was extracted using TRIzol reagent (Invitrogen, CA, USA). In RNase R treatment, RNA (5 μg) was incubated with or without 3 U/μg RNase R (Lucigen, USA) for 15 min at 37 °C. Reverse transcription was performed by using the PrimeScript RT Reagent Kit (TaKaRa, RR037A, Japan). RNA expression was quantified by qRT-PCR with the TB Green Premix Ex Taq II Kit (TaKaRa, RR820, Japan). Meanwhile, we used β-actin, GAPDH, and U6 as internal references for circRNA, mRNA, and miRNA. The primers are listed in the Additional file: Table 1.

### Cell culture and stable transfection

Human gastric mucosal epithelial cell line GES-1 cells and human GC cell lines SNU-16, SNU-1, MKN-45, NCI-N87, and AGS were used. AGS cells were cultured in an F12K medium (Gibco, USA), and the other cells were cultured in RPMI 1640 medium (Gibco, USA). Both media contained 10% fetal bovine serum (FBS) and 1% penicillin-streptomycin. The cells were cultured at 37 °C in a humidified incubator containing 5% CO2. To overexpress circEVI5, full-length circEVI5 cDNA was cloned into the pLC5-ciR vector (Geneseed, Guangzhou, China), and lentivirus was constructed by using 293 T cells. Stable transfection of SNU-1 and MKN-45 cells with circEVI5 or control vector was performed.

### Oligonucleotide transfection

SNU-1 and MKN-45 cells were seeded in a 12-well plate and cultured overnight to 70% confluence before transfection. The miR-4793-3p mimics (25 nM), miR-4793-3p inhibitors (100 nM), and negative control oligonucleotides (RiboBio, Guangzhou, China) were transfected into SNU-1 and MKN-45 cells with Lipofectamine 3000 reagent (Invitrogen, USA) according to the manufacturer’s protocol.

### Cell counting kit-8 (CCK-8) assay

The viability of cells was detected by the CCK-8 assay. A total of 3 000 cells were seeded in 96-well plates and cultured for 24, 48, 72, and 96 h. Subsequently, 10 ul of CCK-8 solution (Dojindo Laboratories, Japan) was added to each well and incubated for 2 h. Then we measured the absorbance value (OD) at 450 nm.

### 5-Ethynyl-20-deoxyuridine (EdU) incorporation assay

The EdU incorporation assay was performed using the BeyoClick EdU-488 Cell Proliferation Kit (Beyotime, Shanghai, China). EdU (10 μM) was added to the cell medium and cultured for 2.5 h. After fixation with 4% paraformaldehyde and permeation with 0.3% Triton X-100, the cells were incubated with a click reaction mixture at room temperature for 30 min in dark. Subsequently, cell proliferation was detected by flow cytometry (BD Biosciences, FACSCanto II, USA).

### PI staining cell cycle assay

For the cell cycle assay, cells were collected and fixed in 75% PBS-diluted ethanol at 4 °C overnight. Then, after washing once with PBS, the cells were resuspended in 200 ul of BD Pharmingen PI/RNase staining buffer (BD Biosciences, #550825, USA) and incubated at room temperature for 15 min. We immediately ran the analysis on a flow cytometer (BD Biosciences, FACSCanto II, USA). The data were analyzed with ModFit software.

### Xenografts in mice

MKN-45 cells transfected with circEVI5 or control vector were harvested and suspended in PBS. Then, we subcutaneously injected the cell suspension into the left-right symmetric lower back of mice (BALB/C nude, 5-6 weeks old, male, *n* = 6). The tumor volume (volume = length × width^2^/2) of the mice was monitored weekly. After 21 days, the mice were euthanized and tumor tissues were excised and weighed. The mouse experiment was conducted in accordance with the ethics guidelines for animal experiments and approved by The Animal Ethical and Welfare Committee of Tianjin Medical University Cancer Institute and Hospital.

### Pull down assay

The biotin-labeled circEVI5 probe was designed and synthesized by GenePharma (Shanghai, China). SNU-1 cells were seeded in a 10 cm dish. When reaching 60–70% density, cells were transfected with the specific circEVI5 probe or control probe for 24 h. After lysis and centrifugation, the supernatant cell lysate was retained and incubated with streptavidin-coupled Dynabeads (C1, Invitrogen) gently in rotation for 30 min at room temperature. Then, the coated beads were washed 2 times with 1X B&W buffer (5 mM Tris-HCl (pH = 7.5), 0.5 mM EDTA, and 1 M NaCl), and binding was completed. Next, RNA was extracted by using the miRNeasy Mini Kit (QIAGEN, Germany), and the cDNA products available for all miRNAs were obtained by the poly(A) tailing reaction (Sangon Biotech, B532451). Finally, we quantified RNA expression by qRT-PCR.

### RNA immunoprecipitation (RIP)

SNU-1 cells were harvested at 90% confluency in a 15 cm dish. The RIP assay was performed using a Magna RIP RNA-Binding Protein Immunoprecipitation Kit (Millipore, USA) according to the manufacturer’s instructions. A human anti-AGO2 antibody was purchased from Millipore. Subsequently, qRT-PCR and agarose gel electrophoresis were carried out to identify the expression of circEVI5 and miRNAs.

### Dual-luciferase reporter assay

A wild-type (circEVI5-WT) or mutant (circEVI5-Mut) fragment of circEVI5 was constructed and inserted into the pmirGLO plasmid (Synbio Tech, Suzhou, China), and a wild-type (FOXO1 3′UTR-WT) or mutant (FOXO1 3′UTR-Mut) fragment of the FOXO1 3′UTR was constructed and inserted into the pmiR-RB-report vector (RiboBio, Guangzhou, China). Then the reporter plasmids and miR-4793-3p mimics or circEVI5 overexpressing lentivirus were co-transfected into HEK-293T cells. Forty-eight hours later, the Dual-Luciferase Reporter System Kit (E1910, Promega, USA) was applied to detect firefly and Renilla luciferase activity.

### RNA fluorescence in situ hybridization (FISH)

A biotin-labeled probe was specific to circEVI5, and a Cy5-labeled probe was specific to miR-4793-3p (Synbio Tech, Suzhou, China). First, AGS and SNU-1 cells were seeded on poly-lysine-coated glass slips, fixed in 4% paraformaldehyde, and permeabilized with 70% ethanol. Then, hybridization was carried out in a hybridization buffer (10% formamide, 10% dextran sulfate, 2×SSC) with circEVI5 probe and miR-4793-3p probe in a 37 °C dark moist chamber overnight. After hybridization, slides were incubated with anti-streptavidin-Alexa Fluor 488(Abcam, USA) at 37 °C for 1 h. Finally, the slides were sealed with tablets containing DAPI (Invitrogen, P36935, USA). All images were acquired on a Zeiss LSM880 confocal microscope system (Leica Microsystems, Germany). The probe sequences are listed in the Additional file: Table 1.

### Western blot

Total protein was extracted using RIPA lysis buffer (Beyotime, Shanghai, China) with a 1% protease inhibitor and phosphatase inhibitor. Twenty-five micrograms of total protein for each sample were separated by SDS-polyacrylamide gel electrophoresis (SDS-PAGE) and transferred onto polyvinylidene fluoride (PVDF) membranes (Millipore). After blocking in 5% nonfat milk at room temperature for 2 h, the membrane was incubated with primary antibody at 4 °C overnight. The next day, the membrane was incubated with HRP-labeled secondary antibody at room temperature for 2 h. Afterward, signals were visualized by ECL chemiluminescent reagent (Millipore). The antibodies were as follows: FOXO1 (1:1000, Proteintech); p21^cip1^ (1:2000, Cell Signaling Technology); p27^kip1^ (1:2000, Cell Signaling Technology); GAPDH (1:3000; Cell Signaling Technology).

### Statistical analysis

All the statistical analyses were performed by SPSS 19.0 (SPSS, Chicago, USA) and GraphPad Prism 8 (GraphPad, CA, USA). Data were calculated by the χ2 or Fisher’s exact test. The Kaplan–Meier curve and log-rank test were used for survival analysis. Date differences between two groups were analyzed by unpaired *t*-test (two-tailed). The correlations were analyzed by Pearson’s test (r, p). The results were shown as the mean ± SD. *P*-value < 0.05 indicated that the difference is statistically significant.

## Supplementary information

Additional file: Table1

## Data Availability

All data generated or analyzed during this study are included in this published article and supplementary information files.
